# Trends in peritoneal surface malignancies: evidence from a Czech nationwide population-based study

**DOI:** 10.1186/s12957-019-1731-4

**Published:** 2019-11-06

**Authors:** Dušan Klos, Juraj Riško, Martin Loveček, Pavel Skalický, Ivana Svobodová, Denisa Krejčí, Bohuslav Melichar, Beatrice Mohelníková-Duchoňová, Radmila Lemstrová

**Affiliations:** 1 Department of Surgery I, Faculty of Medicine and Dentistry, Palacky University Olomouc and University Hospital Olomouc, Olomouc, Czech Republic; 2 Institute of Biostatistics and Analyses, Faculty of Medicine, Masaryk University, Brno, Czech Republic; 3 Institute of Health Information and Statistics of the Czech Republic, Prague, Czech Republic; 4 Department of Oncology, Faculty of Medicine and Dentistry, Palacky University Olomouc and University Hospital Olomouc, Hněvotínská 3, CZ-779 00 Olomouc, Czech Republic; 5 Institute of Molecular and Translational Medicine, Faculty of Medicine and Dentistry, Palacky University Olomouc, Olomouc, Czech Republic

**Keywords:** Incidence, Peritoneal malignancies, Intraperitoneal hyperthermic chemotherapy

## Abstract

**Background:**

The aim of this study is to identify the incidence trends of primary and secondary peritoneal surface malignancies in a representative Czech population.

**Methods:**

Data were obtained from patients registered in the Czech National Cancer Registry between 1979 and 2016. The incidence rates were analyzed between 2012 and 2016. To observe the incidence trends, we analyzed the data from two time periods, 1979–2005 and 2006–2016. The analyzed data included age, sex, and the histological types and primary origins of the malignancies. The Cochrane-Armitage test for linear trends was used for verification of the null hypothesis. The significance level established for hypothesis testing was *p* = 0.05.

**Results:**

Between 2012 and 2016, 230 patients with primary peritoneal tumors were identified and divided into the following groups according to their “International Statistical Classification of Diseases and Related Health Problems, 10th revision” codes: malignant neoplasm of specified parts of the peritoneum (C48.1); malignant neoplasm of the peritoneum, unspecified (C48.2); and malignant neoplasm of overlapping sites of the retroperitoneum and peritoneum (C48.8). Moreover, 549 primary tumors of the appendix (C18.1, encompassing all appendiceal malignancies) and 3137 secondary synchronous peritoneal carcinomatoses of other primary origins were documented. The age-adjusted incidence of primary peritoneal tumors in 2012–2016 was 4.36/year/1,000,000 inhabitants. The age-adjusted incidence of synchronous secondary peritoneal malignancies in 2014–2016 was 99.0/year/1,000,000 inhabitants. The diagnoses of primary peritoneal malignancies followed a stable trend between 1979 and 2016. However, the incidences of primary tumors of the appendix increased by 76.7%.

**Conclusions:**

The data produced in our study ought to clarify the status of peritoneal surface malignancies in the Czech Republic, which can lead to improved planning and development of therapeutic interventions as well as physician training.

## Background

Peritoneal surface malignancies are a very heterogeneous group of diseases with generally poor prognosis [1]. They include both primary malignancies of the peritoneum (such as diffuse malignant peritoneal mesothelioma [DMPM], pseudomyxoma peritonei [PMP], primary peritoneal serous papillary carcinoma, and desmoplastic small round cell tumors) and metastases from other primary tumors (most commonly the colon, stomach, or ovary and less frequently the pancreas, biliary tract, urachus, or urinary tract). The therapeutic approaches and prognoses vary depending on the histological type of primary tumor, the extent of peritoneal involvement, and the patient’s condition and associated comorbidities. A number of papers have been published in recent years highlighting the role of cytoreductive surgery (CRS) and hyperthermic intraperitoneal chemotherapy (HIPEC) in the treatment of peritoneal surface malignancies [2, 3]. CRS and HIPEC have become gold standards in the treatment of DMPM [4] and PMP [5] and are also individually accepted interventions for peritoneal carcinomatosis (PC) of ovarian, colorectal, and gastric origin [6].

Epidemiological data regarding peritoneal surface malignancies are very limited; current data are based on individual national cancer registries and international databases. The most comprehensive data are provided by GLOBOCAN 2008 [7] and other local registries of individual peritoneal surface carcinomas. However, local or specialized registries related to a particular disease provide multicentric data from patients who are already diagnosed and therefore cannot be applied to a population. In the Czech Republic, these data have not been analyzed in detail to date, as diagnoses of peritoneal surface malignancies are very rare and are not published annually in national health reports; therefore, they are not commonly available to the public or to experts.

The aim of this large population-based study was to retrospectively assess the incidence of rare primary peritoneal tumors and secondary PCs of colorectal, ovarian, and gastric origin in the Czech population based on an analysis of data from the Czech National Cancer Registry. Furthermore, we aimed to assess the changes in the trends of diagnosis of peritoneal malignancies over time in terms of diagnostic modalities, pathological staging systems, and surgical practice.

## Methods

Data regarding the number of peritoneal malignancies reported in 1979–2016 as recorded in the Czech National Cancer Registry (CNCR) were retrospectively analyzed. There were three specific analyses performed: the first was of the age-adjusted incidence of primary peritoneal and appendiceal malignancies between the years 2012 and 2016, the second was of trends of primary peritoneal malignancy diagnoses between the years 1979 and 2016 (this subperiod was divided into two groups, 1979–2005 and 2006–2016, to identify the changes and trends in incidence; these periods were artificially proposed to better assess the changes in the trends of diagnosis of peritoneal surface malignancies over time, especially during the last decade), and the third was of incidences of synchronous secondary peritoneal malignancies diagnosed between 2014 and 2016, when reporting such secondary malignancies to the CNCR became mandatory. Cancer registries were introduced in the Czech Republic (then Czechoslovakia) as early as 1951; the CNCR was subsequently established in 1976, with data collection becoming compulsory by law. The CNCR provides summary data for statistical purposes at both the national and international levels and was the source of data for our study. Because our data were extracted from a public database, the study did not require institutional review board approval. Data on peritoneal surface malignancies are documented according to the International Statistical Classification of Diseases and Related Health Problems, 10th revision (ICD-10), which has been in use since 1997. All patients diagnosed with primary or secondary peritoneal malignancies were included. The analyses encompassed malignant neoplasms of specified parts of the peritoneum (C48.1); malignant neoplasms of the peritoneum, unspecified (C48.2); and overlapping lesions of the retroperitoneum and peritoneum (C48.8). Also included were primary tumors of the appendix (C18.1), as these represent the most common source of PMP, as well as secondary peritoneal surface malignancies of other primary origins (most commonly colorectal cancer [CRC], ovarian cancer, and gastric cancer). Diagnostic codes determined by the physician and recorded in each patient’s medical records are compulsorily submitted to the CNCR by the hospital statistical unit. The validity of the data was verified via a comparison with information from the national insurance system, enrollment in which is compulsory in the Czech Republic. The information was rechecked several times and supplemented with any missing post-diagnosis information to ensure that proper statistical analyses were conducted. The incidence of synchronous peritoneal malignancies (metastases) from colorectal, gastric, ovarian, and other cancers has only been compulsorily recorded in the CNCR since 2014. The analyzed data included age, sex, and the histological types and primary origins of the malignancies. The Cochrane-Armitage test for linear trends was used for verification of the null hypothesis. The significance level established for hypothesis testing was *p* = 0.05.

## Results

### Patient demographics

#### Primary peritoneal tumors

**Fig. 1:**
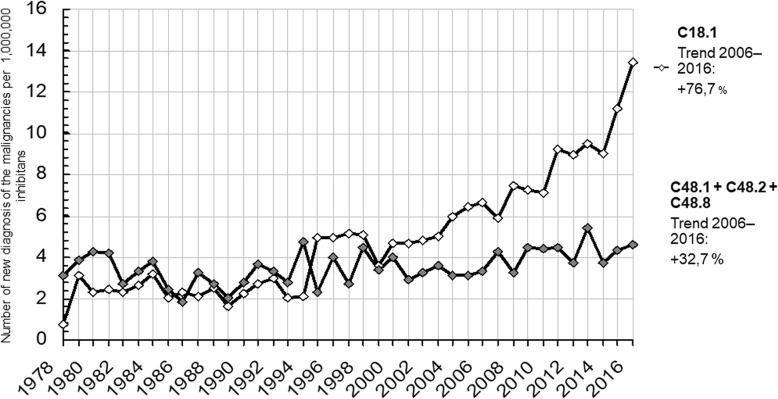
Trends of incidence rates of primary peritoneal (C48.1 + C48.2 + C48.8) and appendiceal (C18.1) malignancies between 1979 and 2016

There were 230 diagnoses of primary peritoneal malignancies, including malignant neoplasm of specified parts of the peritoneum (C48.1); malignant neoplasm of the peritoneum, unspecified (C48.2); and overlapping lesions of the retroperitoneum and peritoneum (C48.8), in the Czech population between 2012 and 2016 (Table 1). The age-adjusted incidence of primary peritoneal tumors was therefore 4.36/year/1,000,000 inhabitants (Fig. 1). Primary peritoneal tumors were significantly more frequent among women than among men (78% vs. 22%; *p* <  0.001) and most commonly manifested in patients aged 60–69 years (34.7%, *p* <  0.001) (Table 2).

**Table 1:** Incidence of primary peritoneal and appendiceal malignancies in the Czech population between 2012 and 2016

	Incidence (number of new cases of malignancies)					
	2012	2013	2014	2015	2016	Total 2012–2016
Malignant neoplasm of the colon [appendix] (C18.1)	94	100	95	118	142	549
Malignant neoplasm of specified parts of the peritoneum (C48.1)	10	27	6	8	10	61
Malignant neoplasm of the peritoneum, unspecified (C48.2)	20	21	24	27	26	118
Overlapping lesion of the retroperitoneum and peritoneum (C48.8)	9	9	9	11	13	51

**Table 2:** Gender and age stratification in the incidence of primary peritoneal and appendiceal malignancies in the Czech population between 2012 and 2016

	Gender			Age by diagnosis					
	Male	Female	*p* rate	0–49 years	50–59 years	60–69 years	70–79 years	80 years and above	*p* rate
Malignant neoplasm of the colon [appendix] (C18.1)	220 (40.1%)	329 (59.9%)	< 0.001	215 (39.2%)	68 (12.4%)	135 (24.6%)	85 (15.5%)	46 (8.4%)	< 0.001
Malignant neoplasm of specified parts of the peritoneum (C48.1)	22 (36.1%)	39 (63.9%)	0.030	9 (14.8%)	11 (18.0%)	18 (29.5%)	15 (24.6%)	8 (13.1%)	0.214
Malignant neoplasm of the peritoneum, unspecified (C48.2)	26 (22.0%)	92 (78.0%)	< 0.001	10 (8.5%)	18 (15.3%)	41 (34.7%)	25 (21.2%)	24 (20.3%)	< 0.001
Overlapping lesion of the retroperitoneum and peritoneum (C48.8)	21 (41.2%)	30 (58.8%)	0.208	3 (5.9%)	8 (15.7%)	18 (35.3%)	12 (23.5%)	10 (19.6%)	0.019

#### Primary tumors of the appendix

A total of 549 primary tumors of the appendix (C18.1), which are the most common source of PMP, were reported in the Czech population between 2012 and 2016 (Table 1); these included all appendiceal malignancies. The age-adjusted incidence was therefore 10.39/year/1,000,000 inhabitants. The database we used does not contain specific information on the incidence of PMP and does not distinguish between epithelial and other types of appendiceal tumors. However, in clinical practice, low-grade appendiceal mucinous neoplasm (LAMN) is classified as a tumor of the appendix (C18.1). Women were diagnosed with tumors of the appendix significantly more frequently than men (59.9% vs. 40.1%; *p* <  0.001) (Table 2); primary tumors of the appendix most often manifested at a younger age, with 39.2% of the diagnoses occurring in patients 0–49 years of age; a second diagnostic peak was observed among patients 60–69 years of age (24.6%), *p* <  0.001 (Table 2).

#### Secondary synchronous peritoneal malignancies

The number of newly diagnosed secondary synchronous peritoneal malignancies, i.e., histologically confirmed metastases in the peritoneum, was 3137 between the years 2014 and 2016 (Table 3). Peritoneal malignancies most commonly occurred in patients with colorectal, ovarian, and gastric neoplasms (861, 489, and 408 cases, respectively). The total numbers of newly diagnosed tumors in 2014–2016 were 23,891 CRCs (including 4645 cases in stage IV with extended metastases), 3051 ovarian cancers (1857 were stages III–IV), 4286 gastric cancers, and 1563 metastases. The age-adjusted incidence of synchronous secondary peritoneal malignancies between 2014 and 2016 was 99.0/year/1,000,000 inhabitants. Synchronous PC was reported in 3.6% of all Czech patients with CRC between 2014 and 2016; 61% of those had ovarian cancer, and 9.5% had gastric cancer. PC less commonly originated from primary tumors of the pancreas, lung, biliary tract, and liver. However, peritoneal spread in these patients invariably accompanied advanced primary tumors and/or other distant metastases, and surgical treatment was rarely, if ever, considered for such patients. Synchronous metastases in CRCs occurred at comparable rates in men and women (49.1 vs. 50.9%; *p* = 0.609) (Table 4). Metastatic peritoneal cancer most commonly occurred in the 60–69 years age group (33.4%; *p* <  0.001) (Table 5).

**Table 3:** Incidence of synchronous peritoneal metastases according to the primary origin in the Czech population between 2014 and 2016

	Incidence (number of new cases of malignancies)					
	2012	2013	2014	2015	2016	Total 2014–2016
Peritoneal carcinomatosis total	–	–	1017	1058	1062	3137
Malignant neoplasm of the colon and rectum (C18–C20)	–	–	269	318	274	861
Malignant neoplasm of the ovary (C56)	–	–	173	167	149	489
Malignant neoplasm of the stomach (C16)	–	–	131	139	138	408
Malignant neoplasm of the pancreas (C25)	–	–	121	117	141	379
Malignant neoplasm of the trachea, bronchus, and lung (C33, C34)	–	–	63	50	64	177
Malignant neoplasm of the gallbladder and biliary tract (C23, C24)	–	–	55	54	58	167
Malignant neoplasm of the liver and intrahepatic bile ducts (C22)	–	–	33	52	39	124
Malignant neoplasm of the uterus (C54, C55)	–	–	34	36	46	116
Other locality of the primary tumor	–	–	138	125	153	416

**Table 4:** Gender stratification in the incidence of synchronous peritoneal metastases according to the primary origin in the Czech population between 2014 and 2016

	Gender		*p* rate
	Male	Female	
Peritoneal carcinomatosis total	1287 (41.0%)	1850 (59.0%)	< 0.001
Malignant neoplasm of the colon and rectum (C18–C20)	423 (49.1%)	438 (50.9%)	0.609
Malignant neoplasm of the ovary (C56)	–	489 (100.0%)	–
Malignant neoplasm of the stomach (C16)	209 (51.2%)	199 (48.8%)	0.621
Malignant neoplasm of the pancreas (C25)	193 (50.9%)	186 (49.1%)	0.719
Malignant neoplasm of the trachea, bronchus, and lung (C33, C34)	112 (63.3%)	65 (36.7%)	< 0.001
Malignant neoplasm of the gallbladder and biliary tract (C23, C24)	63 (37.7%)	104 (62.3%)	0.002
Malignant neoplasm of the liver and intrahepatic bile ducts (C22)	77 (62.1%)	47 (37.9%)	0.007
Malignant neoplasm of the uterus (C54, C55)	–	116 (100.0%)	–
Other locality of the primary tumor	210 (50.5%)	206 (49.5%)	0.845

**Table 5:** Age stratification in the incidence of synchronous peritoneal metastases according to the primary origin in the Czech population between 2014 and 2016

	Age by diagnosis					*p* rate
	0–49 years	50–59 years	60–69 years	70–79 years	80 years and above	
Peritoneal carcinomatosis total	207 (6.6%)	429 (13.7%)	1048 (33.4%)	894 (28.5%)	559 (17.8%)	< 0.001
Malignant neoplasm of the colon and rectum (C18–C20)	56 (6.5%)	103 (12.0%)	262 (30.4%)	257 (29.8%)	183 (21.3%)	< 0.001
Malignant neoplasm of the ovary (C56)	26 (5.3%)	70 (14.3%)	157 (32.1%)	136 (27.8%)	100 (20.4%)	< 0.001
Malignant neoplasm of the stomach (C16)	41 (10.0%)	70 (17.2%)	135 (33.1%)	101 (24.8%)	61 (15.0%)	< 0.001
Malignant neoplasm of the pancreas (C25)	16 (4.2%)	52 (13.7%)	144 (38.0%)	112 (29.6%)	55 (14.5%)	< 0.001
Malignant neoplasm of the trachea, bronchus, and lung (C33, C34)	9 (5.1%)	24 (13.6%)	73 (41.2%)	49 (27.7%)	22 (12.4%)	< 0.001
Malignant neoplasm of the gallbladder and biliary tract (C23, C24)	4 (2.4%)	20 (12.0%)	53 (31.7%)	49 (29.3%)	41 (24.6%)	< 0.001
Malignant neoplasm of the liver and intrahepatic bile ducts (C22)	4 (3.2%)	11 (8.9%)	38 (30.6%)	52 (41.9%)	19 (15.3%)	< 0.001
Malignant neoplasm of the uterus (C54, C55)	4 (3.4%)	13 (11.2%)	44 (37.9%)	36 (31.0%)	19 (16.4%)	< 0.001
Other locality of the primary tumor	47 (11.3%)	66 (15.9%)	142 (34.1%)	102 (24.5%)	59 (14.2%)	< 0.001

### Trends

After dividing the study group into two time periods (1979–2005 and 2006–2016) to better observe the trends, we found that the incidences of primary appendiceal malignancies increased from 3.2 to 6.56 and ultimately to 11.6/year/1,000,000 inhabitants in 1979, 2006, and 2016, respectively. There was a 105% increase between 1979 and 2005 as well as another 76.7% jump between 2006 and 2016. The incidences of primary peritoneal malignancies (i.e., ICD-10 codes C48.1, C48.2, and C48.8) remained stable within the two time periods, decreasing from 4.0 to 3.16/year/1,000,000 inhabitants between 1979 and 2005 and then increasing to 4.2/year/1,000,000 inhabitants between 2006 and 2016. (Fig. 1).

## Discussion

Peritoneal surface malignancies represent a very heterogeneous group of diseases that are usually diagnosed at an advanced stage when they are already metastatic. Advanced-stage tumors are usually considered incurable and terminal, a view that also applies to primary tumors of the peritoneal surface. Since the early 1990s, systemic therapies including CRS and HIPEC have been increasingly advancing. In the cases of PMP and DMPM, CRC and HIPEC should be the first-choice intervention because there are no surgical, radiotherapeutic, or other treatments that produce comparable results [8, 9]. Moreover, systemic treatments of peritoneal malignancies are still mostly based on cytotoxic agents, while targeted treatment options are limited [10]. As the role of the immune system in the outcomes of neoplastic disorders is increasingly being recognized [11, 12], and since cellular populations responsible for immune responses are present in the peritoneal cavity [13–16], the activation of the host response may represent an important HIPEC mechanism.

Epidemiological data describing peritoneal malignancies are very poor, as they represent 0.08% of all newly diagnosed malignancies in the Czech Republic annually. Hence, the management and outcomes of these malignancies in our population remain relatively unclear, as is also the case in Western Europe and the USA.

### Increasing diagnosis rates of peritoneal malignancies

In 2012–2016, the age-adjusted incidence rate of primary peritoneal tumors (ICD-10 groups C48.1, C48.2, and C48.8) was 4.36 cases per 1,000,000 inhabitants per year. Most of these patients had primary malignant peritoneal mesothelioma. Based on the Surveillance, Epidemiology, and End Results program as well as the European Cancer Incidence and Mortality data [17, 18], age-adjusted incidence rates among men range from 0.5 to approximately 3 cases per 1,000,000 per year. Approximately 2500 new patients with mesothelioma are registered each year in the USA. The higher numbers in our country compared to worldwide data can be explained by the fact that asbestos was a common and almost indispensable building component in the Czech Republic during the communist era; these were gradually reconstructed in 1990–2000. Moreover, there were only minimal regulations for protection against radon radiation until recently. Asbestos and radon exposure are considered risk factors for the development of DMPM [19].

The age-adjusted incidence of appendiceal malignancies in the Czech population averages 10.39/year/1,000,000 inhabitants. A significant increase in the incidence (76.7%) was apparent between 2006 and 2016. These malignancies include both epithelial and other (e.g., carcinoid) malignancies. These data reflect the rarity of appendiceal epithelial lesions, as also evidenced by the results of a Dutch study group [20] that reported the incidence of appendiceal epithelial lesions at approximately 9/year/1,000,000. The incidence of PMP, which most frequently originates from an appendiceal neoplasm, is even lower at an estimated 2 per 10,000 laparotomies or 1/year/1,000,000 population, with a predominance among women (2–3-fold more frequent than in men) [21, 22]. The estimated incidence of PMP in the Czech population is approximately 10–11 cases/year. The difference in the number of newly diagnosed cases of appendiceal tumors between 2012 and 2016, 94 vs. 142, can be explained by the introduction of a compulsory national screening program for CRCs in 2014, including occult bleeding detection programs for individuals 45 years of age and over, as well as colonoscopy screenings.

Secondary malignancies represent a very heterogeneous group of tumors, and curative treatment options in many cases are minimal. However, CRS and HIPEC have curative potentials in select patients with PC originating from colorectal, ovarian, and gastric cancers. The primary drawback when diagnosing secondary PCs is the relatively low specificity and sensitivity of imaging methods [23]. The small size of the tumor deposits (typically less than 1 cm) negatively affects sensitivity [24]. Furthermore, the peritoneal spread of tumor cells characteristically follows the anatomical outline of normal abdominal structures, rendering radiologic detection even more challenging. Thus, the presence of PC is usually only detected during laparotomy for primary tumor resection. The underestimation of PC by preoperative imaging, combined with the aforementioned lack of awareness, likely explains the near absence of data on the incidence of PC. Synchronous PC was present in 3.6% of all Czech patients with CRC diagnosed between 2014 and 2016, as well as in 9.5% of all patients with gastric cancer. Two population-based studies found that the incidences of synchronous PCs that developed from CRC in Dutch and Swedish patients were 4.8% and 4.3%, respectively [25, 26]. Risk factors for developing PC include a right-sided tumor, advanced T-stage, advanced N-stage, poor differentiation grade, and younger age at the time of diagnosis [27]. Isolated PCs in patients with CRC are rare, and most patients have metastases at other (non-PC) sites, specifically the liver and lungs. The best available clinical data are available in multi-institutional registries [28, 29] but require careful interpretation because surgeons’ experiences, techniques, and perioperative care protocols differ widely between institutions [30]. However, median survival rates of up to 63 months have been reported following CRS and HIPEC, with limited postoperative morbidity and mortality [31]; this suggests that treatment should be considered in all patients with isolated PC secondary to CRC.

### Age and sex

We found that the age-specific peak incidence of primary peritoneal tumors was 60–69 years (34.7%; *p* < 0.001) and that women were more frequently affected than men (78% vs. 22%; *p* < 0.001). The incidence of primary appendiceal malignancies was also significantly higher in women than in men (59.9% vs. 40.1%; *p* < 0.001), and our study showed two age-specific peak incidences for this disease: 0–49 years (39.2%) and 60–69 years (24.6%; *p* < 0.001). Synchronous metastases in colorectal tumors occur at comparable frequencies in men and women (49.1% vs. 50.9%; *p* = 0.609). Moreover, metastatic peritoneal cancer is most frequent among individuals 60–69 years of age (33.4%; *p* < 0.001); the incidence of this secondary malignancy in the elderly increased, which is concordant with the general increase in the rates of all malignancies due to aging.

### Could the pathological scoring system have led to increased diagnoses?

Our analysis of the trends of peritoneal surface malignancy diagnoses led to our investigation of the reasons for the increasing incidence rates, most notably in appendiceal malignancies. Although all types of appendiceal malignancies were examined, the rates of the increases between 1979 and 2005 (105%) and between 2006 and 2016 (76.7%) cannot be explained only by the numbers of tumors. Rather, changing the pathological classification of appendiceal malignancies plays an important role. The classification of these tumors is still the subject of debate owing to their unique biological behavior. Appendiceal neoplasms frequently lack overtly malignant features such as infiltrative invasion but can nevertheless spread to the peritoneum. Peritoneal lesions are generally histologically bland, with rare lymph node or hematogenous metastases, but PMP is often ultimately fatal [32]. It has also been recommended that the term “pseudomyxoma peritonei” should either be used as a clinical diagnosis or be abandoned completely [33]. Many of these tumors were previously diagnosed as a group of malignancies with uncertain or unknown behavior (ICD-10: D37.3 and D48.4, neoplasm of uncertain or unknown behavior of the appendix and peritoneum). In 2010, the World Health Organization (WHO) introduced the Classification of Tumors of the Digestive System [34], which recognized three main categories of mucinous neoplasm: mucinous adenoma, LAMN, and appendiceal adenocarcinoma. Another pathological classification system was introduced by the Peritoneal Surface Oncology Group International (PSOGI) [35]. The PSOGI classification of the PMP peritoneal component was further divided into three categories: low-grade PMP is characterized by abundant extracellular mucin with scant strips or small islands of simple-to-focally proliferative epithelium, with minimal cytologic atypia and rare mitoses; high-grade PMP, which exhibits more abundant cellularity with moderately-to-poorly differentiated carcinomatous patterns; and signet ring cell (SRC) PMP, which indicates the presence of any SRC component. Acellular mucin is classified separately. Primary appendiceal lesions are categorized as (1) LAMN, (2) high-grade appendiceal mucinous neoplasm, (3) mucinous adenocarcinoma, (4) adenocarcinoma with SRC, and (5) SRC carcinoma [35]. Introducing this classification into clinical practice has led to improvements in diagnosis and interpretation of appendiceal malignancies, but the primary importance of these classification systems is the prognostic stratification of patients with peritoneal malignancies predicting their response to treatment and survival. However, it remains unclear whether the PSOGI classification provides better prognostic stratification than the current WHO classification [36].

### Are increasing diagnosis rates due to changes in diagnostic modalities and surgical practice?

Another important factor to consider regarding the increase in the incidence of peritoneal surface malignancies is the evolution in diagnostic and surgical practices. PMP and appendiceal malignancies, as well as other peritoneal surface malignancies, are characterized by minimum clinical symptoms. Most patients are diagnosed only in very advanced stages, presenting with symptoms of malnutrition, cachexia, ascites, and bowel movement disorders. The use of imaging methods, especially ultrasonography and computed tomography (which are used routinely for diagnosing various symptoms of abdominal discomfort), leads to an increase in the detection of pathological conditions in the abdominal cavity. Among women, endovaginal ultrasonography is a critical component of gynecological screening. The introduction of percutaneous biopsies and diagnostic laparoscopy, or of laparoscopic procedures in general, has led to a further increase in the numbers of diagnosed peritoneal malignancies. In the Czech Republic, the introduction of colonoscopy screening in high-risk groups since 2009 has been critical for increasing the detection of appendiceal malignancies.

### Estimated numbers of patients undergoing CRS and HIPEC

Based on our population-based study, the estimated number of patients eligible for CRS and HIPEC in the Czech Republic is approximately 162 per year (20 with PMP, 46 with DMPM, and 96 with PC/CRC, which is approximately one third of the 287 patients diagnosed annually excluding patients with other malignancies, particularly of the liver and lungs, and patients with comorbidities limiting surgical procedures). Approximately 18 procedures per year were performed in the Czech Republic between 2012 and 2016. Thus, only 11% of patients received adequate treatment, although the estimated cost of palliative systemic treatment for DMPM (cisplatin and pemetrexed) is many-fold higher than that for CRS and HIPEC. Owing to the complexity of the procedure, the learning curve for surgeons to perform this type of procedure is estimated at 140–220 patients [37]. The number of procedures in global high-volume centers as indicated in the literature varies between 24 and 123 procedures per year, with an average of 55 [30]. With an estimated annual number of 162 patients and an ideal figure of 55 procedures per center per year, 3 centers per 10 million population would be required in the Czech Republic.

### Strengths and limitations

There were several limitations to this study. As a retrospective review, it was prone to information bias, and there may have been missing or ambiguous data collected. Furthermore, the population-based data lacked information regarding the use of systemic chemotherapy, surgical therapy, CRS, and HIPEC for patients with peritoneal malignancies. There was also no central pathology review, which may have led to marked variations in diagnoses among pathologists of different institutions in the Czech Republic. Some data may have been missing because of unknown ICD-10 classifications. However, owing to the mandatory reporting required to the CNCR, the data are very valuable and our study presents a unique population-based overview regarding the incidences and trends of peritoneal surface malignancies.

## Conclusions

Our results show a stable trend in the incidence of rare primary peritoneal tumors within the Czech population. We observed a significant increase in primary appendiceal tumors, possibly owing to earlier detection as part of the nationwide colonoscopy screening program and the use of ultrasonography and computed tomography of the abdominal cavity in daily practice. The increased incidence may also be due to the markedly improved pathological classifications of these malignancies. However, current treatment guidelines do not distinguish between these malignancies, and CRS and HIPEC are still not the curative standard intervention for patients with peritoneal surface malignancies in the Czech Republic, except PMP and DMPM. Moving forward, our epidemiological data and discovered trends can help modify our treatment practice and increase the proportions of patients who undergo CRS and HIPEC in our region.

## Data Availability

All data generated or analyzed during this study are included in this published article (and its supplementary information files).

## Abbreviations

CNCR: Czech National Cancer Registry
CRC: Colorectal cancer
CRS: Cytoreductive surgery
DMPM: Diffuse malignant peritoneal mesothelioma
HIPEC: Hyperthermic intraperitoneal chemotherapy
ICD-10: International Statistical Classification of Diseases and Related Health Problems, 10th revision
LAMN: Low-grade appendiceal mucinous neoplasm
PC: Peritoneal carcinomatosis
PMP: Pseudomyxoma peritonei
PSOGI: Peritoneal Surface Oncology Group International
WHO: World Health Organization
